# Benefits of nitric oxide administration during cardiopulmonary bypass on postoperative outcomes in adult patients: a meta-analysis and systematic review

**DOI:** 10.1186/s12871-026-03707-0

**Published:** 2026-02-25

**Authors:** Yanting Zhang, Mingming Wang, Gang Chen, Youfa Zhou

**Affiliations:** 1 Department of Anesthesiology, Sir Run Run Shaw Hospital, School of Medicine, Zhejiang University, Qingchun East Road No. 3, Hangzhou, 310020 China; 2 Department of Anesthesiology, The First Affiliated Hospital of Zhejiang Chinese Medical University (Zhejiang Provincial Hospital of Chinese Medicine), Hangzhou, China

**Keywords:** Nitric Oxide, Post - operative Complications, Cardiopulmonary Bypass

## Abstract

**Background:**

Nitric oxide (NO) is reported to play several protective roles in the inflammatory response and ischemia/reperfusion injury. This study evaluated the effect of nitric oxide (NO) on postoperative outcomes in adults undergoing cardiac surgery with cardiopulmonary bypass (CPB).

**Methods:**

We systematically searched PubMed, Web of Science, and EMBASE (inception–April 2025) for relevant RCTs. Two reviewers independently screened studies, extracted data, and assessed risk of bias (Cochrane RoB 1) and evidence certainty (GRADE). Meta-analysis was performed using random-effects models.

**Results:**

Eight RCTs involving 774 patients were finally included. The use of NO did not reduce the postoperative mortality (RR 0.71, 95% CI 0.36 to 1.42), the duration of postoperative ventilation (MD -0.23 h, 95% CI -1.18 to 0.71), the length of stay in hospital (MD -0.07 days, 95% CI -0.55 to 0.42) or in intensive care unit (MD -4.17 h, 95% CI -8.74 to 0.4). This study demonstrated that NO administration significantly reduced serum levels of creatine kinase-MB at both 24 h (MD -19.36 ng/ml, 95% CI -28.72 to -10.00) and 48 h (MD -17.47 ng/ml, 95% CI -19.53 to -15.41) postoperatively, though it did not affect troponin levels. Concurrently, NO was associated with a significantly lower incidence of postoperative acute kidney injury (RR 0.78, 95% CI 0.64 to 0.93), but not with a reduced need for renal replacement therapy (RR 0.88, 95% CI 0.45 to 1.72).

**Conclusions:**

This meta-analysis of RCTs did not demonstrate a clear benefit of nitric oxide administered via cardiopulmonary bypass on mortality, duration of mechanical ventilation, or length of stay in adult cardiac surgery. Although suggestive renal and cardioprotective signals were observed, these findings are preliminary and require confirmation in larger, methodologically rigorous trials before definitive conclusions can be drawn.

**Trial registration:**

INPLASY2022120002.

**Supplementary Information:**

The online version contains supplementary material available at 10.1186/s12871-026-03707-0.

## Introduction

Cardiopulmonary bypass (CPB) remains a cornerstone technique in cardiac surgery, temporarily assuming the functions of the heart and lungs. However, this procedure is intrinsically associated with several pathophysiological challenges. The contact of blood with the non-physiological surfaces of the CPB circuit can trigger endothelial injury, a systemic inflammatory response, and coagulopathy. These effects are further compounded by the surgical trauma itself and subsequent ischemia-reperfusion injury, collectively contributing to postoperative morbidity. Notably, this inflammatory cascade is a key driver of low cardiac output syndrome (LCOS), a complication linked to prolonged mechanical ventilation, adverse long-term outcomes, and increased mortality [1].

Nitric oxide (NO) has emerged as a molecule of significant interest due to its potential protective properties. Evidence suggests that NO can mitigate inflammatory responses and ameliorate ischemia-reperfusion injury [2, 3]. Furthermore, it plays a pivotal role in modulating the interplay between endothelial dysfunction and inflammation [4]. In recent years, the therapeutic administration of NO directly into the CPB circuit has been investigated in cardiac surgery, yielding inconsistent clinical results. Several studies have reported benefits, including a cardioprotective effect [5–7], a reduced incidence of acute kidney injury and LCOS [8, 9], and shorter durations of invasive mechanical ventilation and intensive care unit (ICU) stay [7]. Conversely, other trials, while supporting some myocardial protection, found no significant improvement in broader clinical outcomes [5, 6, 9]. Some research has even concluded that NO delivery via CPB offers no clinical benefit [10]. A recent large multicenter trial in pediatric patients reinforced this null finding, demonstrating no significant increase in ventilator-free days or reduction in LCOS, ICU, or hospital stay with NO administration [11]. A prior systematic review attempted to synthesize evidence on this topic but was limited by the inclusion of only three pilot randomized controlled trials (RCTs) [12]. A subsequent meta-analysis focused on pediatric cardiac surgery concluded that exogenous NO via CPB provided no postoperative benefits [13]. Given the substantial physiological and surgical differences between adult and pediatric populations, the effects observed in children cannot be directly extrapolated to adults. To date, no dedicated meta-analysis has comprehensively evaluated the efficacy of NO administration via CPB specifically in adult cardiac surgery. With the recent publication of additional relevant studies in adults, a timely and focused systematic review and meta-analysis is imperative to clarify its role in this distinct patient population.

Therefore, the aim of this study was to conduct a systematic review and meta-analysis to determine the efficacy of NO administration during CPB in adult patients undergoing cardiac surgeries. We hypothesized that there would be no benefit on mortality, duration of mechanical ventilation or length of ICU and hospital stay, but potentially benefits on myocardial protection.

## Materials and methods

### Search strategy and trial selection

This study was conducted in accordance with the Preferred Reporting Items for Systematic Reviews and Meta-analyses (PRISMA) reporting guideline [14] and prospectively registered in the international platform of registered systematic review and meta-analysis protocols (INPLASY), and the registration information is available at https://inplasy.com (registration number: INPLASY2022120002). Neither ethical approval nor patients’ consent was needed as this study was a secondary analysis of published data. A systematic literature search was performed across three major electronic databases: PubMed, Web of Science, and Embase. The search strategy employed subject terms and keywords related to “Nitric Oxide” and “Cardiopulmonary Bypass” without restrictions on publication language. The search encompassed all records from database inception until April 1, 2025. To ensure comprehensiveness, the reference lists of all included articles and relevant review papers were manually screened for additional eligible studies.

This meta-analysis included randomized controlled trials (RCTs) that enrolled adult patients (≥ 18 years) undergoing cardiac surgery with CPB. The intervention of interest was the administration of nitric oxide directly into the CPB circuit, compared against a control group receiving a placebo or standard CPB management without NO. To minimize clinical and methodological heterogeneity, studies were excluded if the primary intervention involved the administration of any NO-donor drugs, as these represent distinct pharmacological and delivery approaches.

The process of study selection was consistent with the description in our previous study [15]. The citations obtained from the searches mentioned previously were added to Endnote (Thomson Reuters version X7). After duplications were detected and excluded, two independent reviewers initially evaluated the titles and abstracts of the citations, followed by a full-text assessment. Disagreements between reviewers were resolved by a third party.

### Data extraction, synthesis, and measurement of outcomes

As described in the previous study [15], the data extraction process was carried out by two independent reviewers using a standardized form. In case of disagreement, a third reviewer was consulted to make the final decision. The numerical data reported in tables or figures served as the primary source for all the data. When the data was presented in graphical form only, we used a graph digitizing software (Engauge digitizer 10.8, Mark Mitchel, 2014) to extract the data. In case of insufficient data, we made an effort to contact the corresponding authors of the studies.

The characteristics extracted from the included studies were the author’s name, year of publication, location of the study, sample size of participants, their age and sex, intervention and comparator details, and the duration of CPB. We also extracted measures of clinical outcomes most commonly used in recent publications. The primary outcomes of this analysis were all-cause mortality at the longest available follow-up and the duration of postoperative invasive mechanical ventilation (hours). Secondary outcomes encompassed several markers of postoperative morbidity and resource utilization. These included postoperative levels of myocardial injury biomarkers (Creatine Kinase-MB [CK-MB] and cardiac troponin I [cTnI]), the incidence of acute kidney injury (AKI), the need for renal replacement therapy (RRT), intensive care unit length of stay (ICU LOS), and hospital length of stay (hospital LOS). Additionally, we documented the specific definition criteria for Acute Kidney Injury (AKI) used in each included study (e.g., KDIGO, RIFLE, or other criteria).

### Quality assessment

The methodological quality and risk of bias of the included randomized controlled trials were independently assessed by two reviewers using the Cochrane Collaboration’s risk of bias tool (RoB 1). This tool systematically evaluates potential bias across seven key domains: (1) random sequence generation (2), allocation concealment (3), blinding of participants and personnel (4), blinding of outcome assessment (5), completeness of outcome data (6), selective outcome reporting, and (7) other potential sources of bias. For each domain, the risk was judged and categorized as ‘low’, ‘high’, or ‘unclear risk’ according to the standard criteria outlined in the Cochrane Handbook.

The overall certainty of the evidence for the primary outcomes was evaluated using the Grading of Recommendations, Assessment, Development, and Evaluations (GRADE) framework. The evidence was appraised across five domains: risk of bias (study limitations), consistency, directness, precision, and publication bias. The assessments were performed independently by two authors. Any discrepancies in judgement were resolved through consensus discussion or, if necessary, by consulting a third reviewer [15]. GRADEpro software [GRADEpro GDT: GRADEpro Guideline Development Tool (Software), McMaster University, 2020] was used to prepare the Summary of findings (SoF) table.

### Statistical analysis

All statistical analyses were conducted using Review Manager software (RevMan, version 5.4.1; The Cochrane Collaboration, Copenhagen, Denmark). Given the anticipated clinical and methodological heterogeneity among the included studies, a DerSimonian and Laird random-effects model was employed to calculate pooled effect estimates [16]. For continuous outcomes, the inverse variance method was used to derive study weights, while the Mantel-Haenszel method was applied for dichotomous outcomes. Treatment effects are presented as mean differences (MDs) with 95% confidence intervals (CIs) for continuous data and risk ratios (RRs) with 95% CIs for dichotomous data. When necessary, means and standard deviations (SDs) were estimated from available statistics (e.g., medians, ranges, or standard errors) using the established methods described by Luo and colleagues [17] to allow for quantitative synthesis.

We employed the *I²* statistic to quantify heterogeneity, which estimates the proportion of total variation across studies attributable to heterogeneity. When the *I*² statistic exceeded 50%, indicating substantial heterogeneity, we then conducted subgroup analyses to investigate potential sources of this variation [18]. Subgroup analysis regarding the timing and duration of nitric oxide administration was performed to investigate heterogeneity. The timing and duration of iNO therapy were categorized into two subgroups: NO administration throughout the entirety of the CPB process; NO administration during operation and continuing to the post-operative stage. Subgroup analyses will be performed if ≥ 3 studies meet the criteria for each subgroup; otherwise, subgroup analyses will be omitted, and the rationale will be reported. Moreover, sensitivity analyses were performed: (i) restricted to studies with low risk of bias, to assess the influence of methodological quality on the robustness of the pooled estimates; and (ii) excluding studies in specialized populations (e.g., patients with pulmonary hypertension, or undergoing left ventricular assist device–related procedures), to evaluate the generalizability of the findings. The funnel plot was used to assess publication bias if more than ten studies were included. For all tests, a two-tailed *P* value < 0.05 was considered statistically significant.

## Results

### Search results and studies included

**Fig. 1:**
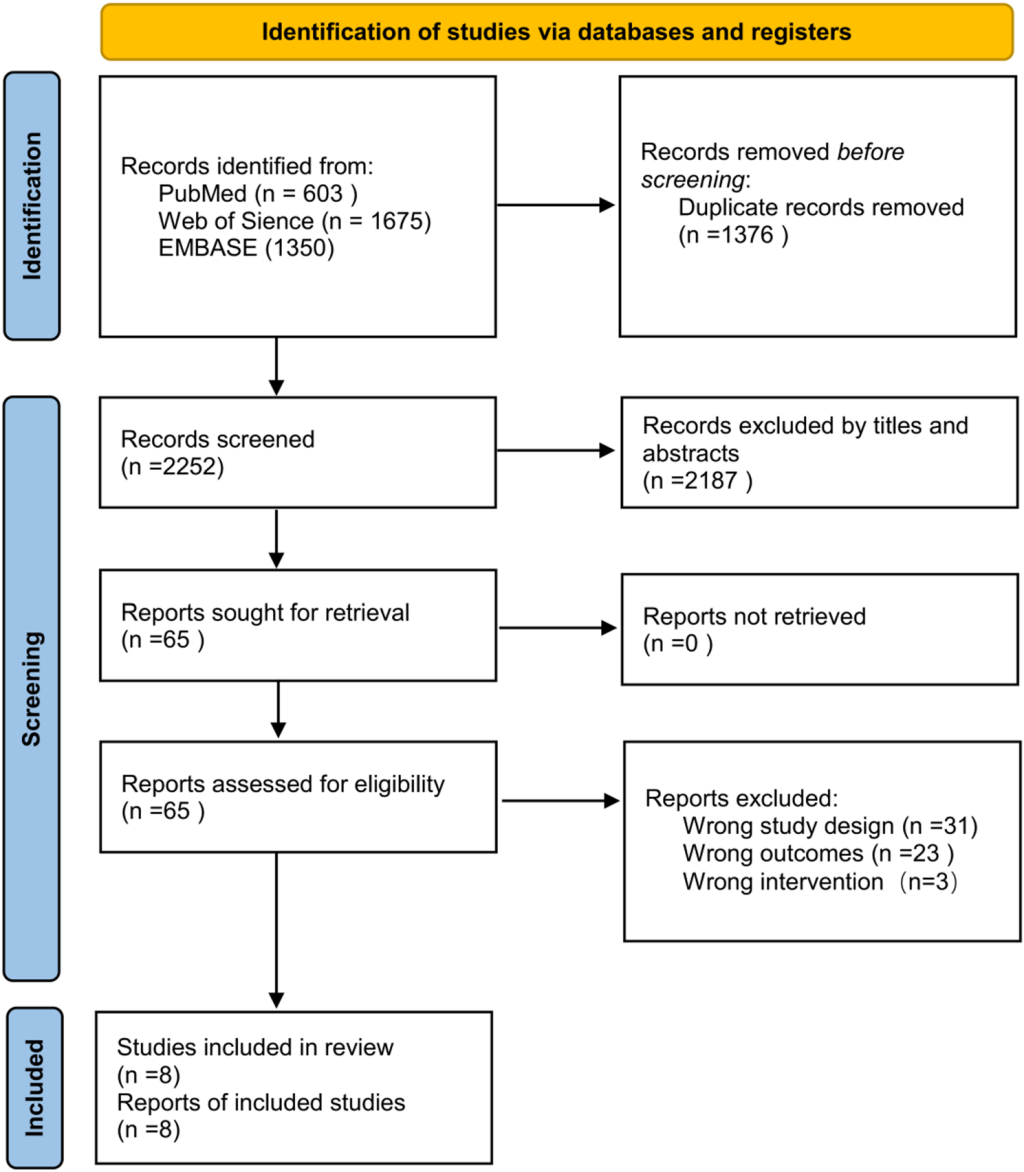
The flowchart of the article selection process

The initial literature search identified 3628 potentially relevant articles from all databases. After removing duplicate studies, 2252 articles were screened by titles and abstracts. Full-text review was conducted for 65 studies and finally 8 studies were included in the analysis based on the inclusion and exclusion criteria. The flowchart of the article selection process is shown in Fig. 1.

### Description of included studies

The characteristics of included studies were shown in Table 1. A total of 774 participants were included in the 8 RCTs (NO group: 380; control group 394). Among these, six studies [5, 8, 19–22] were conducted in patients undergoing standard cardiac surgery (i.e., coronary artery bypass grafting and/or valve surgery), one study [23] enrolled patients with pulmonary hypertension, and one study [24] was performed in patients undergoing left ventricular assist device implantation. NO was administered solely during surgery in four studies [5, 8, 21, 22], whereas its use extended into the postoperative period in the remaining four studies [19, 20, 23, 24]. The NO-treated group received 40 ppm of NO via the extracorporeal circulation in four studies [5, 8, 22, 24], 20 ppm of NO in three studies [19, 21, 23] and 80 ppm of NO in one study [20].

**Table 1:** Characteristic of included studies

Study ID	Country	Population	Patients number		Gender (F/M)		Age (Mean ± SD)		Intervention		CPB time (Mean ± SD/median [IQR])	
			NO	CON	NO	CON	NO	CON	NO	CON	NO	CON
Gianetti 2004 [19]	Italy	Patients received aortic valve replacement combined with aortocoronary bypass	14	15	5/9	7/8	70 ± 13	69 ± 10	20 ppm during and for 8 h after CPB	Standard CPB	107 ± 40	110 ± 35
Potapov 2011 [24]	US	Patients received LVAD placement	73	77	9/64	12/65	57.6± 9.75	54.01 ± 1.95	40 ppm from weaning attempt from CPB and continued until extubated or for 48 h	Standard CPB	Unavailable	Unavailable
Lei 2018 [20]	China	Patients received Heart valve surgeries	117	127	65/52	75/52	48.4 ± 8.6	48.7 ± 9.5	80 ppm during and for 24 h after CPB	Standard CPB	Unavailable	Unavailable
Kamenshchikov 2019 [5]	Russia	Patients received CABG	30	30	5/25	9/21	61.4 ± 6.4	58.7 ± 6.2	40 ppm gNO during CPB	Standard CPB	110.9 ± 39.9	111.4 ± 31.7
Pichugin 2020 [21]	Russia	Patients received heart valve surgery and combined procedures	30	30	17/13	18/12	58.6 ± 1.4	54.1 ± 1.4	20 ppm during the entire operation	Standard CPB	104.8 ± 33.2	98.6 ± 37.1
Nakane 2021 [23]	Japan	Patients developing pulmonary hypertension during weaning from extracorporeal circulation	30	65	11/19	25/40	72.8 ± 9.5	73.6 ± 8.1	20–40 ppm from the weaning from CPB to extubation	Standard CPB	248 ± 86	247 ± 103
Kamenshchikov 2022 [8]	Russia	Patients received CABG and valve surgeries	48	48	18/30	17/31	64 ± 7.8	63 ± 8.1	40-ppm gNO during CPB	Standard CPB	Unavailable	Unavailable
Azem 2024 [22]	Israel	Patients received CABG, valvular or combined surgeries	51	47	14/37	10/37	64.0 ± 11.1	69.0 ± 8.1	40 ppm during CPB	Standard CPB	112 [81 to 130]	101 [89 to 121]

*NO* Nitric oxide, *CON* Control group, *CPB* Cardiopulmonary bypass, *LVAD* Left Ventricular Assist Device, *CABG *Coronary Artery Bypass Grafting

### Risk of bias

**Fig. 2:**
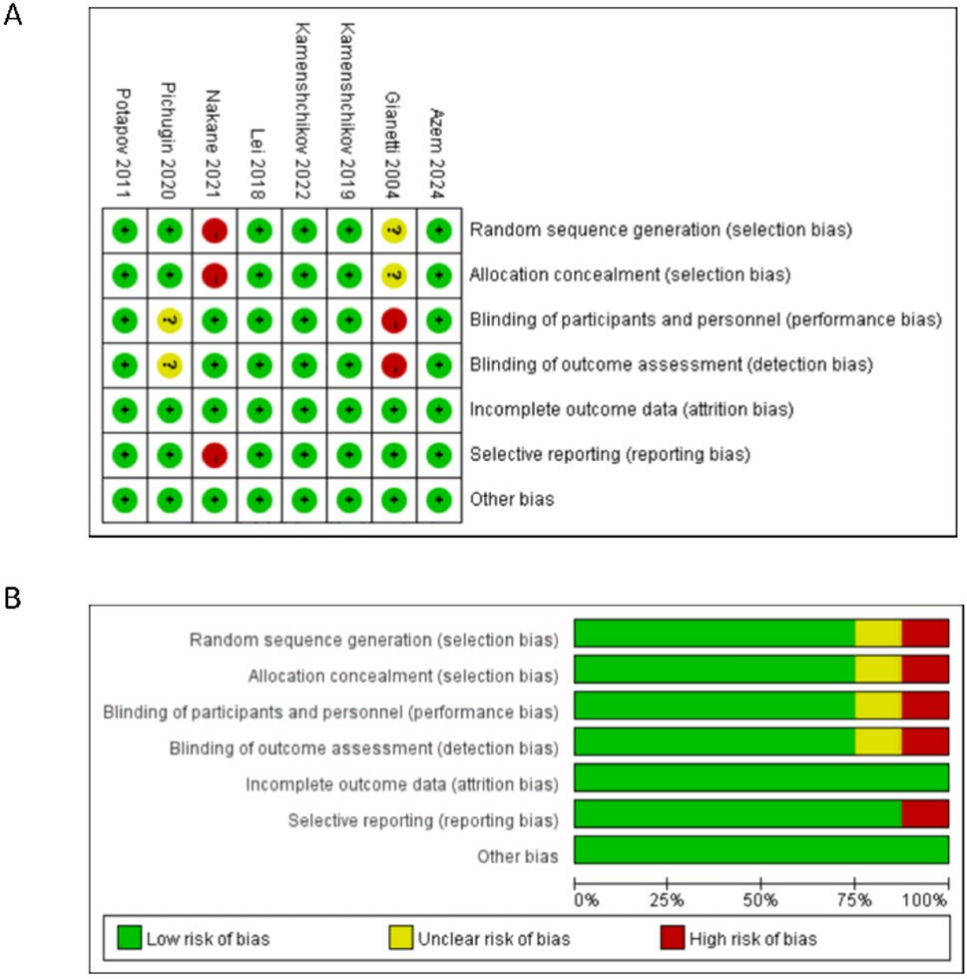
Quality assessment of included studies. The green circles indicate lack of bias; red circles indicate the presence of bias. **A** Risk of bias for each included study. **B** The overall summary of bias of the included studies

The results of bias risk assessment were presented in Fig. 2. Two studies were considered to be at potential high risk of bias because of inadequate or incorrect randomization and insufficient blinding [19, 23]. All the other domains of included were at low risk of bias.

### Outcomes

#### Mortality

**Fig. 3:**
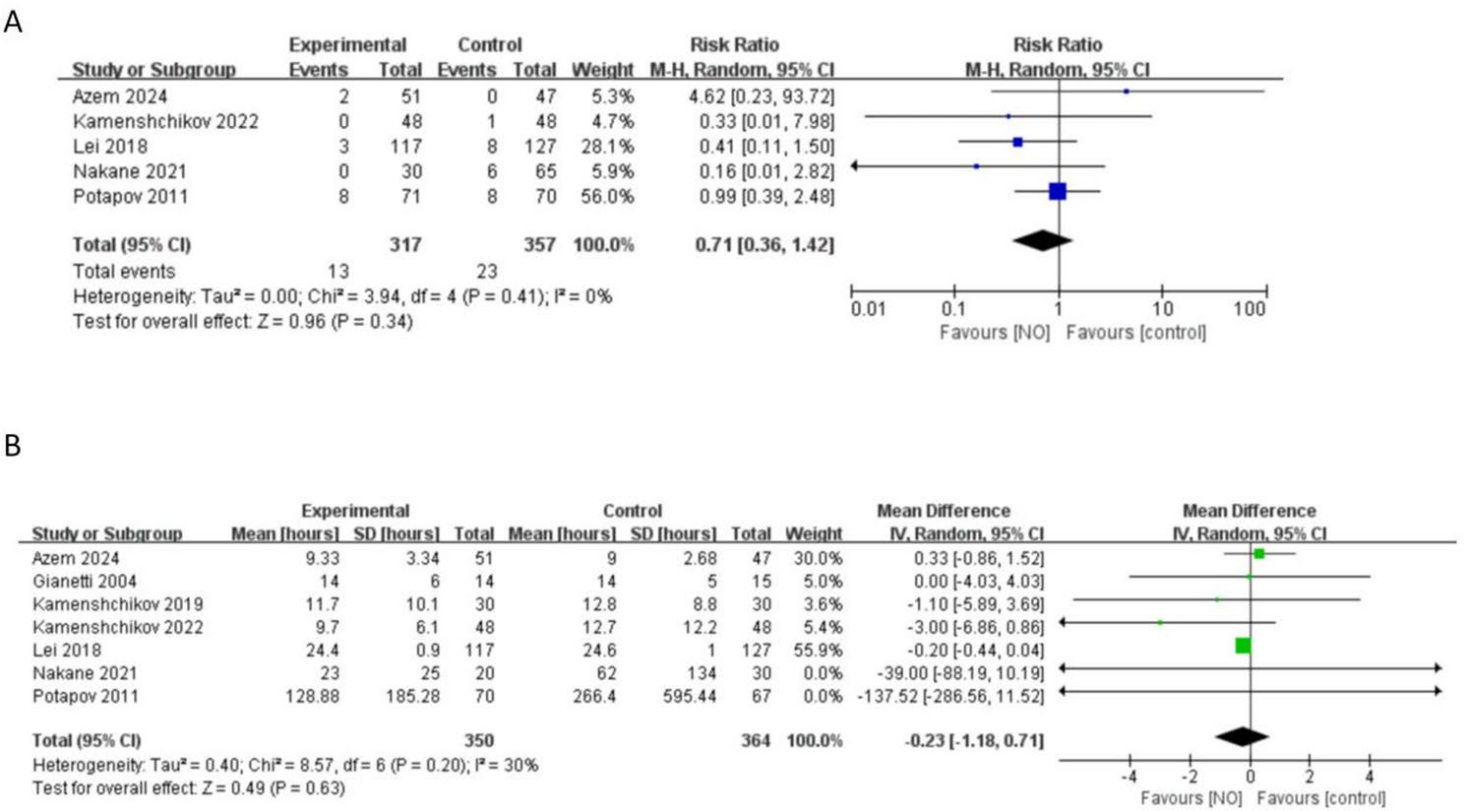
Forest plot diagram of meta-analysis of (**A**) mortality and (**B**) duration of postoperative mechanical ventilation (hours)

Five studies (*n* = 674 ) reported mortality at the longest follow-up [8, 20, 22–24]. NO administration did not significantly reduce the mortality among the studies (RR 0.71, 95% CI 0.36 to 1.42, *I*^*2*^ = 0%, *P* = 0.34) (Fig. 3A).

#### Duration of postoperative mechanical ventilation

Seven included studies (*n* = 714) reported the duration of postoperative mechanical ventilation [5, 8, 19, 20, 22–24]. Meta-analysis of these studies showed that NO did not significantly influence the duration of postoperative mechanical ventilation (MD -0.23 h, 95% CI -1.18 to 0.71, *I*^*2*^ = 30%, *P =* 0.63) (Fig. 3B).

#### Markers of myocardial injury

**Fig. 4:**
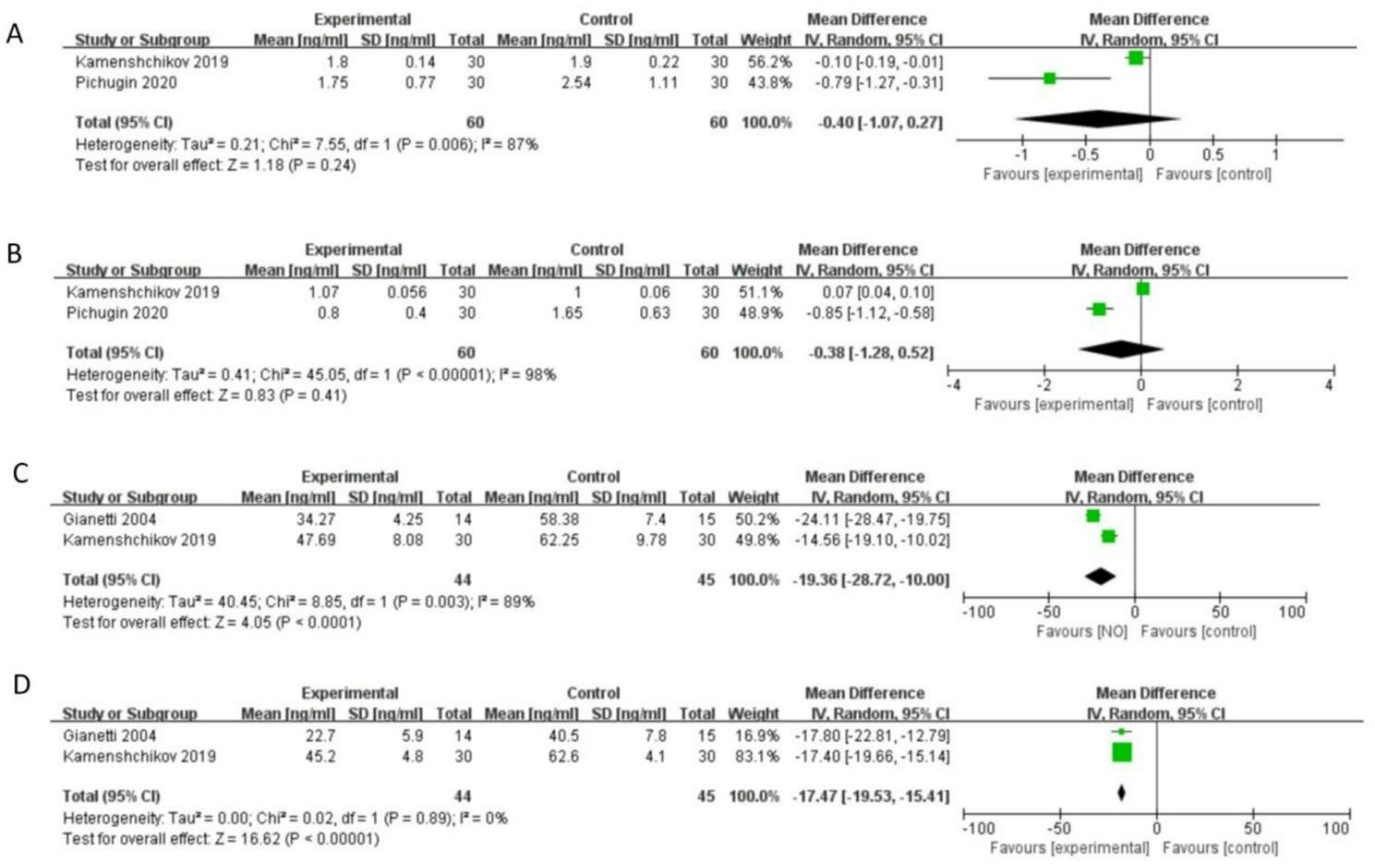
Forest plot diagram of meta-analysis of: (**A**) cardiac troponin I at 24 h (ng/mL); (**B**) cardiac troponin I at 48 h (ng/mL); (**C**) CK-MB at 24 h (ng/mL); (**D**) CK-MB at 48 h (ng/mL) postoperatively

The levels of cardiac troponin I at different time points after surgery were reported in two studies [5, 21]. No significant difference was observed between the two groups in levels of cardiac troponin I at 24 h (*n* = 120, MD -0.40 ng/ml, 95% CI -1.07 to 0.27, *I*^*2*^ = 87%, *P* = 0.24), and 48 h (*n* = 120, MD -0.38 ng/ml, 95% CI -1. 28 to 0.52, *I*^*2*^ = 98%, *P* = 0.41) postoperatively (Fig. 4A and B).

However, NO significantly reduced the level of CK-MB at 24 h (*n* = 89, MD -19.36 ng/ml, 95% CI -28.72 to -10.00, *I*^*2*^ = 89%, *P* < 0.05), and 48 h (*n* = 89, MD -17.47 ng/ml, 95% CI -19.53 to -15.41, *I*^*2*^ = 0%, *P* < 0.05) postoperatively from the meta-analysis of two studies [5, 19] (Fig. 4C and D).

#### Incidence of acute kidney injury (AKI) and needs for renal replacement therapy (RRT)

**Fig. 5:**
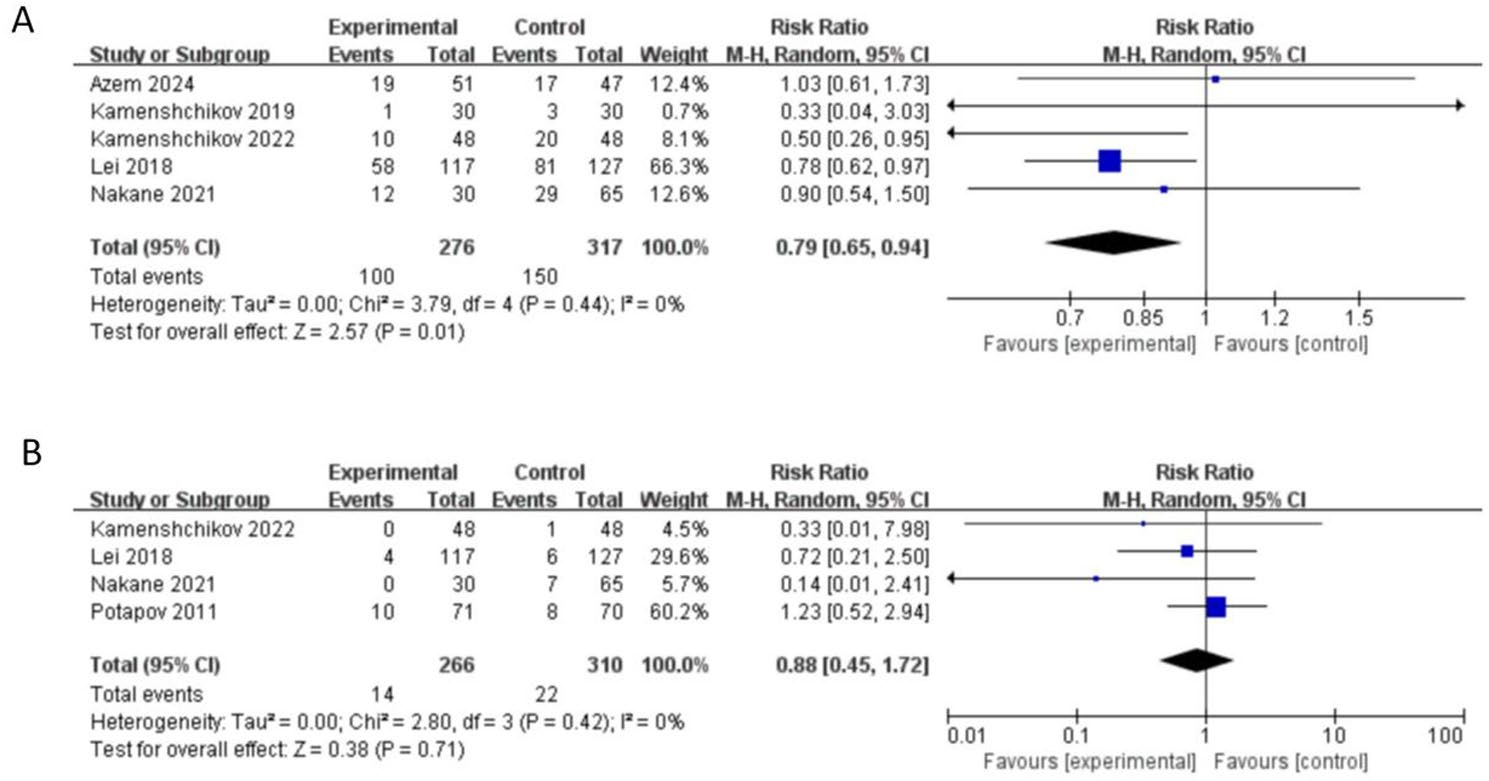
Forest plot diagram of meta-analysis of incidence of AKI (**A**) and needs for RRT (**B**)

The incidence of AKI, as defined by the KDIGO criteria was reported in five studies [5, 8, 20, 22, 23] (*n* = 593) and NO was associated with a significant lower risk of postoperative acute kidney injury (AKI) based on meta-analytic results (RR 0.79, 95% CI 0.65 to 0.94, *I*^*2*^ = 0%, *P <* 0.05)(Fig. 5A) .

Four studies (*n* = 576) reported the needs for postoperative renal replacement therapy [8, 20, 23, 24]. There was no significant difference between two groups in the needs for renal replacement therapy (RR 0.88, 95% CI 0.45 to 1.72, *I*^*2*^ = 0%, *P* = 0.71) (Fig. 5B).

#### Length of stay (LOS) in hospital and ICU

**Fig. 6:**
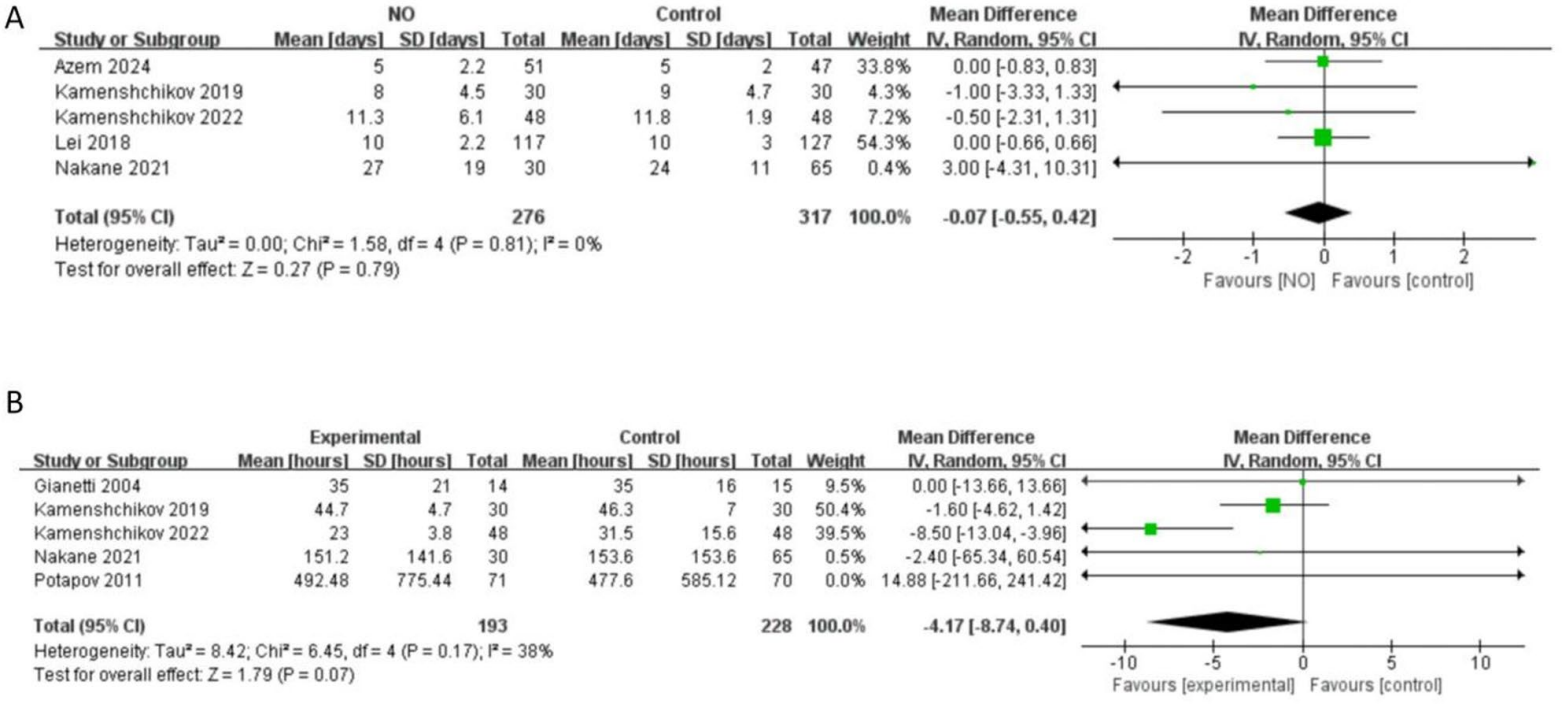
Forest plot of the meta-analysis of length of stay: (**A**) hospital stay (days); (**B**) ICU stay(hours)

There five studies [5, 8, 20, 22, 23] measured the LOS in hospital and no difference in LOS in hospital was observed (*n* = 593, MD -0.07 days, 95% CI -0.55 to 0.42, *I*^*2*^ = 0%, *P* = 0.79) (Fig. 6A). A meta-analysis of five studies [5, 8, 19, 23, 24] revealed no statistically significant difference in ICU length of stay between the two patient groups (*n* = 421, MD -4.17 h, 95% CI -8.74 to 0.4, *I*^*2*^ = 38%, *P =* 0.07) (Fig. 6B).

### Subgroup and sensitivity analyses

In most meta-analyses, the heterogeneity among studies, as determined by the *I²* statistic, was not substantial. Several analyses focusing on myocardial injury biomarkers exhibited significant heterogeneity; however, due to the limited number of included studies (*n* < 3), subgroup analyses could not be conducted.

Moreover, sensitivity analyses—both excluding studies with high risk of bias [19, 23] and excluding studies involving specialized populations such as those with pulmonary hypertension or those undergoing left ventricular assist device (LVAD)-related procedures [23, 24] —did not materially alter the study results, including mortality, duration of postoperative mechanical ventilation, incidence of acute kidney injury and need for renal replacement therapy, as well as length of stay in hospital and the intensive care unit ( Supplementary Table 1).

### Quality of evidence

The certainty of evidence for each outcome, assessed using the GRADE framework, is summarized in Table 2. For mortality (5 RCTs, *n* = 674), the evidence was rated as moderate, downgraded one level for imprecision because the 95% confidence interval around the pooled effect included both no effect and appreciable benefit or harm (relative risk reduction or increase > 25%). For duration of postoperative mechanical ventilation (7 RCTs, *n* = 714) and length of hospital stay (5 RCTs, *n* = 593), the evidence was rated as high, with no serious concerns across any GRADE domains. For length of ICU stay (5 RCTs, *n* = 421), the evidence was rated as moderate, downgraded one level for imprecision; the 95% confidence interval crossed the threshold for a clinically important difference (SMD < − 0.5) and included no effect. For incidence of acute kidney injury (5 RCTs, *n* = 593), the evidence was rated as moderate, downgraded one level for publication bias, as published evidence was limited to a small number of trials (*n* = 5) and three of them showed benefit. For need for renal replacement therapy (4 RCTs, *n* = 576), the evidence was rated as moderate, downgraded one level for imprecision; the confidence interval included both no effect and appreciable benefit or harm (RRR or RRI > 25%). For cardiac troponin I at 24 and 48 h (2 RCTs each, *n* = 120), the evidence was rated as low, downgraded two levels owing to serious inconsistency (significant heterogeneity across studies) and serious imprecision (total population size < 400). For CK‑MB at 24 h (2 RCTs, *n* = 89), the evidence was rated as very low, downgraded one level each for risk of bias (only two studies included, one of which was at high risk of bias), inconsistency (significant heterogeneity), and imprecision (total population size < 400). For CK‑MB at 48 h (2 RCTs, *n* = 89), the evidence was rated as very low, downgraded for risk of bias (as above), imprecision (total population size < 400), and publication bias (only two small trials available, both reporting benefit).

**Table 2:** Quality assessment of evidence

Quality assessment							Summary of Findings				
Participants (studies) Follow up	**Risk of bias**	**Inconsistency**	**Indirectness**	**Imprecision**	**Publication bias**	**Overall quality of evidence**	**Study event rates (%)**		**Relative effect** (95% CI)	**Anticipated absolute effects**	
							**With control**	**With NO**		**Risk with control**	**Risk difference with NO** (95% CI)
Mortality											
674 (5 studies)	no serious risk of bias	no serious inconsistency	no serious indirectness	serious^1^	undetected	⊕⊕⊕⊝ **MODERATE** ^1^ due to imprecision	23/357 (6.4%)	13/317 (4.1%)	**RR 0.71** (0.36 to 1.42)	**Study population**	
										**64 per 1000**	**19 fewer per 1000** (from 41 fewer to 27 more)
										**Moderate**	
										**63 per 1000**	**18 fewer per 1000** (from 40 fewer to 26 more)
Duration of postoperative mechanical ventilation (Better indicated by lower values)											
714 (7 studies)	no serious risk of bias	no serious inconsistency	no serious indirectness	no serious imprecision	undetected	⊕⊕⊕⊕ **HIGH**	364	350	**-**		The mean duration of postoperative mechanical ventilation in the intervention groups was **0.23 lower** (1.18 lower to 0.71 higher)
Troponin I 24 h postoperatively (Better indicated by lower values)											
120 (2 studies)	no serious risk of bias	serious^2^	no serious indirectness	serious^3^	undetected	⊕⊕⊝⊝ **LOW** ^2,3^ due to inconsistency, imprecision	60	60	**-**		The mean troponin i 24 h postoperatively in the intervention groups was **0.4 lower** (1.07 lower to 0.27 higher)
Troponin I 48 h postoperatively (Better indicated by lower values)											
120 (2 studies)	no serious risk of bias	serious^2^	no serious indirectness	serious^3^	undetected	⊕⊕⊝⊝ **LOW** ^2,3^ due to inconsistency, imprecision	60	60	**-**		The mean troponin i 48 h postoperatively in the intervention groups was **0.38 lower** (1.28 lower to 0.52 higher)
CK-MB 24 h postoperatively (Better indicated by lower values)											
89 (2 studies)	serious^4^	serious^2^	no serious indirectness	serious^3^	undetected	⊕⊝⊝⊝ **VERY LOW** ^2,3,4^ due to risk of bias, inconsistency, imprecision	45	44	**-**		The mean ck-mb 24 h postoperatively in the intervention groups was **19.36 lower** (28.72 to 10 lower)
CK-MB 48 h postoperatively (Better indicated by lower values)											
89 (2 studies)	serious^4^	no serious inconsistency	no serious indirectness	serious^3^	reporting bias strongly suspected ^5^	⊕⊝⊝⊝ **VERY LOW** ^3,4,5^ due to risk of bias, imprecision, publication bias	45	44	**-**		The mean ck-mb 48 h postoperatively in the intervention groups was **17.47 lower** (19.53 to 15.41 lower)
Incidence of AKI											
593 (5 studies)	no serious risk of bias	no serious inconsistency	no serious indirectness	no serious imprecision	reporting bias strongly suspected ^6^	⊕⊕⊕⊝ **MODERATE** ^6^ due to publication bias	150/317 (47.3%)	100/276 (36.2%)	**RR 0.79** (0.65 to 0.94)	**Study population**	
										**473 per 1000**	**99 fewer per 1000** (from 28 fewer to 166 fewer)
										**Moderate**	
										**417 per 1000**	**88 fewer per 1000** (from 25 fewer to 146 fewer)
Needs for RRT											
576 (4 studies)	no serious risk of bias	no serious inconsistency	no serious indirectness	serious^7^	undetected	⊕⊕⊕⊝ **MODERATE** ^7^ due to imprecision	22/310 (7.1%)	14/266 (5.3%)	**RR 0.88** (0.45 to 1.72)	**Study population**	
										**71 per 1000**	**9 fewer per 1000** (from 39 fewer to 51 more)
										**Moderate**	
										**78 per 1000**	**9 fewer per 1000** (from 43 fewer to 56 more)
Length of stay in hospital (Better indicated by lower values)											
593 (5 studies)	no serious risk of bias	no serious inconsistency	no serious indirectness	no serious imprecision	undetected	⊕⊕⊕⊕ **HIGH**	317	276	**-**		The mean length of stay in hospital in the intervention groups was **0.07 lower** (0.55 lower to 0.42 higher)
Length of stay in ICU (Better indicated by lower values)											
421 (5 studies)	no serious risk of bias	no serious inconsistency	no serious indirectness	serious^8^	undetected	⊕⊕⊕⊝ **MODERATE** ^8^ due to imprecision	228	193	**-**		The mean length of stay in icu in the intervention groups was **4.17 lower** (8.74 lower to 0.4 higher)

^1^ 95% confidence interval suggests no effect, while a relative risk reduction is greater than 25%

^2^ Significant heterogeneity

^3^ Total population size is less than 400

^4^ Only two studies were included, and one of them had a high risk of bias

^5^ Published evidence is limited to a small number of trials (*n* = 2),and all of which are showing benefits of the studied intervention

^6^ Published evidence is limited to a small number of trials (*n* = 5),and 3 of wihch are showing benefits of the studied intervention

^7^ 95% confidence intervalaround the pooledestimate of effect includes both (1) no effect and (2) appreciable benefit a relative risk reduction (RRR) and relative risk increase (RRI) greater than 25%

^8^ 95% confidence interval includes both no effect and a clinically important effect (crosses the SMD threshold of -0.5)

## Discussion

In this study, we systematically evaluated the impact of nitric oxide (NO) administration via cardiopulmonary bypass on postoperative outcomes in adult patients undergoing cardiac surgery. Based on the current available evidence, it appears that the administration of NO via cardiopulmonary bypass does not significantly reduce the postoperative mortality, duration of postoperative mechanical ventilation incidence, levels of cardiac troponin I 24 h and 48 h postoperatively, the needs for renal replacement therapy or the LOS in hospital and ICU. However, NO significantly reduced the incidence of postoperative AKI, the levels of CK-MB 24 h and 48 h after surgery.

Our analysis of NO administration in adult cardiac surgery patients did not find a significant benefit on several key clinical outcomes. These results are consistent with a previous meta-analysis, which included both pediatric and adult patients, reported reduced ICU stay and mechanical ventilation duration in children but not in adults [25]. Similarly, the large NITRIC trial in pediatric patients which was not included by the previous meta-anlysis also found no benefit in ventilator-free days or other hard clinical endpoints [11]. This may be attributed to the multi-factorial nature of these major clinical outcomes, which is influenced by factors beyond the scope of NO’s physiological effects, such as preoperative comorbidities, surgical complexity, and postoperative management.

Consistent with our hypothesis, a preliminary and notable finding from this meta-analysis is the significant reduction in postoperative levels of CK-MB at both 24 and 48 h following surgery. This observed reduction in a surrogate biomarker may suggest a potential cardioprotective role of NO, the mechanisms of which could speculatively include improving mitochondrial efficiency in cardiac muscle cells, exerting a favorable modulation on plasma membrane ion channels, and supporting the electrical syncytium of the heart tissue [3]. However, it is crucial to interpret this finding with caution. The clinical relevance of this isolated biomarker change remains uncertain, as we did not observe a consistent reduction in troponin I levels, nor was the analysis powered to assess hard clinical endpoints (e.g., major adverse cardiac events).The discrepancy between biomarkers may reflect differences in their sensitivity and specificity, or variations in NO administration protocols across studies.

Most importantly, the robustness of the CK-MB finding is materially limited. The results exhibit significant statistical heterogeneity, likely attributable to variability in NO dosing, timing, and surgical type. Due to the inclusion of only two studies, it was not possible to perform subgroup analyses or meta-regression to investigate the sources of this heterogeneity. Consequently, the pooled estimate is unstable and should be considered exploratory. Therefore, this signal, while hypothesis-generating, requires confirmation from a larger number of methodologically homogeneous studies designed to evaluate clinically meaningful outcomes.

We observed a significant trend toward reduced AKI with NO use. The renoprotective mechanism may involve counteracting CPB-induced renal medullary hypoxia and vasoconstriction [26], scavenging of free hemoglobin and restoration of NO bioavailability [27, 28] and attenuation of Damage-Associated Molecular Pattern (DAMP)-mediated injury [29, 30]. However, the lack of a significant reduction in renal replacement therapy suggests that NO may attenuate subclinical AKI without preventing more severe renal dysfunction.

The observed benefits in specific biochemical markers (e.g., CK-MB) and organ-specific outcomes (e.g., AKI incidence) without corresponding improvements in hard clinical endpoints highlight the complexity of NO’s physiological effects. Variability in NO dosing (20, 40 or 80 ppm), timing (intraoperative only vs. extended postoperative use), surgical type (CABG, Valve surgeries or left ventricular assist device implantation) and patient selection (e.g., baseline hypoxemia or renal function) may contribute to the heterogeneity of results. For example, Azem et al. [22] found that patients with baseline hypoxemia derived significant benefit from NO, suggesting that targeted administration in high-risk subgroups may be more effective.

To assess the robustness of the pooled estimates, we performed sensitivity analyses excluding studies at high risk of bias and, separately, excluding studies involving specialized populations such as those with pulmonary hypertension or those undergoing left ventricular assist device (LVAD)-related procedures. For all outcomes where such analyses were feasible, the direction and magnitude of the effect remained stable, reinforcing confidence in the primary findings.

Although the inclusion of clinically heterogeneous populations introduces a degree of indirectness—which should be considered when extrapolating these results to specific patient subgroups—the sensitivity analyses suggest that our main conclusions are not disproportionately driven by any single study or population type.

However, for the four key myocardial injury biomarker outcomes (e.g., CK-MB at 24 h), formal sensitivity analyses were not feasible. This was either because no studies rated as high risk of bias contributed to the pooled estimate, or because the meta‑analysis was based on only two studies. Accordingly, these specific findings should be interpreted with caution, and their robustness warrants confirmation in future studies with larger, more homogeneous cohorts.

According to the GRADE approach, the certainty of evidence for the outcomes in this study varied from low to high. The limited number of studies, small sample sizes, and variability in protocols preclude definitive conclusions. Therefore, future studies should focus on implementing standardized intervention protocols, enlarging sample sizes, employing rigorous blinding designs, and using uniformly defined core outcome measures to generate high-quality evidence. Encouragingly, the ongoing NORISC trial (NCT06702553), a large-scale, multicenter study planning to enroll 3,650 participants, is specifically designed to investigate whether NO administration leads to reduced intensive support in patients undergoing cardiac surgery with cardiopulmonary bypass. Future such trials should focus on high-risk populations and standardized NO administration regimens to better elucidate which patients are most likely to benefit from this intervention, and the completion of this ongoing study will provide further high-quality evidence on this topic.

## Conclusions

The present systematic review and meta-analysis of RCTs found that administration of nitric oxide via cardiopulmonary bypass was not associated with significant reductions in postoperative mortality, duration of mechanical ventilation, or length of hospital and ICU stay in adult cardiac surgery patients. However, signals of potential renal and cardioprotective effects were observed, as reflected by reductions in the incidence of acute kidney injury and in postoperative CK-MB levels. These findings should be interpreted as hypothesis-generating rather than conclusive. Given the methodological limitations, the small number of studies for key biomarker outcomes, and the substantial heterogeneity identified, further high-quality, adequately powered trials are warranted to confirm these potential benefits and to establish their clinical relevance.

## Supplementary Information

Supplementary Material 1.

## Data Availability

All data analysed during this study are included in this published article and its supplementary information files.

## Abbreviations

NO: Nitric oxide
CPB: Cardiopulmonary bypass
RCTs: Randomized controlled trials
LOS: Length of stay
CK-MB: Creatine Kinase-MB
cTnI: Cardiac troponin I
AKI: Incidents of acute kidney injure
RRT: Renal replacement therapy
