# Supplementary material for: Benefits of nitric oxide administration during cardiopulmonary bypass on postoperative outcomes in adult patients: a meta-analysis and systematic review
Source: BMC Anesthesiol. 2026 Feb 25;26:206. doi: 10.1186/s12871-026-03707-0 (PMC13041255; doi:10.1186/s12871-026-03707-0)
Supplement: Supplementary file 1 — Supplementary Material 1. [file 12871_2026_3707_MOESM1_ESM.docx]

**Supplementary Table 1. Results of Sensitivity Analysis Excluding studies with Specialized Populations and high risk of bias**

| **Outcome** | **Analysis** | **No. of Studies** | **Pooled Effect (95% CI)** | ***P*** | ***I²*** | **Direction Change** |
| --- | --- | --- | --- | --- | --- | --- |
| Mortality | Main analysis (All studies) | 5 | 0.71 [0.36, 1.42] | 0.34 | 0% |  |
|  | Sensitivity analysis  (Excluding studies with specialized populations) | 3 | 0.60 [0.16, 2.17] | 0.43 | 11% | Unchanged |
|  | Sensitivity analysis (Excluding studies with high risk of bias) | 4 | 0.78 [0.38, 1.59] | 0.5 | 0% | Unchanged |
| Duration of postoperative mechanical ventilation | Main analysis (All studies) | 7 | -0.23 hours [-1.18, 0.71] | 0.63 | 30% |  |
|  | Sensitivity analysis  (Excluding studies with specialized populations) | 5 | -0.19 hours [-0.42, 0.04] | 0.11 | 0% | Unchanged |
|  | Sensitivity analysis (Excluding studies with high risk of bias) | 5 | -0.22 hours [-1.16, 0.71] | 0.64 | 35% | Unchanged |
| Incidence of acute kidney injury | Main analysis (All studies) | 5 | 0.79 [0.65, 0.94] | 0.01 | 0% |  |
|  | Sensitivity analysis  (Excluding studies with specialized populations) | 4 | 0.77 [0.59, 0.99] | 0.04 | 15% | Unchanged |
|  | Sensitivity analysis (Excluding studies with high risk of bias) | 4 | 0.77 [0.59, 0.99] | 0.04 | 15% | Unchanged |
| Incidence of needs for renal replacement therapy | Main analysis (All studies) | 4 | 0.88 [0.45, 1.72] | 0.71 | 0% |  |
|  | Sensitivity analysis  (Excluding studies with specialized populations) | 2 | 0.65 [0.21, 2.07] | 0.47 | 0% | Unchanged |
|  | Sensitivity analysis (Excluding studies with high risk of bias) | 3 | 0.98 [0.49, 1.96] | 0.95 | 0% | Unchanged |
| Length of stay in hospital | Main analysis (All studies) | 5 | -0.07 days [-0.55, 0.42] | 0.79 | 0% |  |
|  | Sensitivity analysis  (Excluding studies with specialized populations) | 4 | -0.08 days [-0.56, 0.41] | 0.75 | 0% | Unchanged |
|  | Sensitivity analysis (Excluding studies with high risk of bias) | 4 | -0.08 days [-0.56, 0.41] | 0.75 | 0% | Unchanged |
| Length of stay in intensive care unit | Main analysis (All studies) | 5 | -4.17 hours [-8.74, 0.40] | 0.07 | 38% |  |
|  | Sensitivity analysis  (Excluding studies with specialized populations) | 3 | -4.16 hours [-9.80, 1.48] | 0.15 | 69% | Unchanged |
|  | Sensitivity analysis (Excluding studies with high risk of bias) | 3 | -4.78 hours [-10.96, 1.41] | 0.13 | 68% | Unchanged |
